# Multidisciplinary treatment combined with a modified apically positioned flap technique for generalized stage III grade C periodontitis: A five-year follow-up case report

**DOI:** 10.1097/MD.0000000000042037

**Published:** 2025-05-23

**Authors:** Shun Mao, Hui Xie, Fei Ma, Chang Zeng, Jian Liu, Jincai Guo, Yan Xie

**Affiliations:** a School of Stomatology, Hunan University of Chinese Medicine, Changsha, China; b Changsha Stomatological Hospital, Changsha, China.

**Keywords:** a modified apically positioned flap, peri-implant keratinized mucosa, periodontal surgery, periodontitis, soft tissue augmentation surgery

## Abstract

**Rationale:**

The management of patients with generalized stage III grade C periodontitis involves complex considerations, particularly in cases of inadequate peri-implant keratinized mucosa. We present a case study highlighting the multidisciplinary management of periodontitis and the feasibility of a modified apically positioned flap procedure to increase the width of the keratinized mucosa at the implant site during the second stage of implant surgery.

**Patient concerns:**

A 45-year-old woman presented to our hospital with a 4-year history of tooth mobility that caused difficulty chewing and bleeding gums when brushing.

**Diagnoses:**

The patient was diagnosed with generalized stage III grade C periodontitis.

**Interventions:**

The patient underwent a initial periodontal therapy, periodontal surgery, endodontic treatment, implant treatment, prosthodontic treatment, soft tissue augmentation procedures during the second stage of implant surgery, and supportive periodontal therapy.

**Outcomes:**

The patient showed a healthy periodontal status and stable occlusal function after 5 years of follow-up.

**Lessons:**

Multidisciplinary management of generalized stage III grade C periodontitis requires full consideration of systematic periodontal therapy, personalized treatment planning, and patient compliance; in particular, a modified apically positioned flap procedure may serve as an option for soft tissue augmentation at the dental implant site.

## 1. Introduction

Periodontitis is a chronic and inflammatory disease caused by plaque microorganisms that leads to the irreversible destruction of the periodontal tissues. This condition not only increases the risk of tooth loss,but can also result in masticatory dysfunction, which affects both physical and mental health of patients.^[1]^ Global epidemiological studies suggest that approximately 50% of the world’s population is affected by periodontitis, with severe cases reaching a prevalence as high as 11%.^[2,3]^

Treatment plans for severe periodontitis with residual periodontal pockets after initial nonsurgical treatment typically involve periodontal surgery. This includes thorough debridement of root surfaces and furcation areas under visualization to restore the morphology of the hard and soft tissues around the periodontium or restorations.^[4]^ Clinical studies have shown that guided tissue regeneration (GTR) is an effective method of restoring missing periodontal tissue, especially in areas such as angular bone defects and molar furcation involvement.^[5–7]^

Dentition defects are common in patients with severe periodontitis, and implant treatment can effectively restore the morphology and occlusal function of the defective dentition with positive long-term outcomes.^[8]^ However, research indicates that insufficient peri-implant keratinized mucosa (KM) may lead to increased peri-implant mucosal recession and marginal bone resorption, thereby increasing the risk of peri-implantitis.^[9,10]^ Furthermore, delayed implant treatment can exacerbate the lack of peri-implant KM and affect the long-term survival of implant treatment.^[11,12]^ Therefore, proper management of the peri-implant soft tissue is crucial in preventing peri-implantitis.^[13]^ Tavelli et al^[14]^ demonstrated that apically positioned flaps have become one of the most effective techniques for increasing the width of the KM, regardless of the soft tissue graft material used in combination. Additionally, Bassetti et al^[15]^ found that the second stage of implant surgery is an important period for soft tissue remodeling, ultimately enhancing the predictability of the final restorative outcome.

Otherwise, recent research indicates that the integration of immediate implant placement with immediate restoration, alongside soft or hard tissue augmentation techniques, is common in the maxillary anterior esthetic zone. de Siqueira et al^[16]^ found that this approach effectively reduced horizontal bone resorption and stabilized gingival margins over 4 years. Further research confirms that it significantly improves buccal bone thickness, maintains gingival levels, reduces tissue resorption and improves esthetic and functional outcomes.^[17]^ Furthermore, a systematic review of 12 studies from 2012 to 2023 evaluated the esthetic risks and effectiveness of immediate implant placement and immediate restoration in the context of buccal plate defects in the maxillary anterior region. It found that thinner buccal bone plates increased the risk of gingival recession, especially in thin gingival biotypes undergoing flap surgery, resulting in greater interproximal recession. However, the incorporation of soft tissue augmentation, such as connective tissue grafting, significantly improves gingival stability and esthetic outcomes.^[18]^ In particular, the importance of using minimally invasive approaches for peri-implant soft tissue augmentation has been highlighted. A retrospective study showed that the use of tunneling techniques with connective tissue grafting, as opposed to flapless immediate implant placement with autogenous bone graft in the buccal gap, significantly improved soft tissue esthetics and reduced gingival recession.^[19]^ These studies highlight the growing maturity of immediate implant placement with immediate restoration and emphasize the importance of soft tissue and bone grafting for long-term stability and esthetics. Selection of appropriate indications, use of minimally invasive techniques and management of tissue grafting are critical to long-term esthetic and functional success.We present a case study highlighting the multidisciplinary management of periodontitis and the use of a modified apically positioned flap procedure to increase the width of the KM at the implant site during the second stage of implant surgery.

## 2. Case presentation

A 45-year-old woman presented to our hospital with complaints of tooth mobility that had been present for 4 years, causing difficulty in chewing and bleeding gums when brushing. Despite receiving 6 months of scaling and root planing treatment at a dental clinic without improvement, she was referred to the General Dental Department for further systemic treatment. Her medical history was unremarkable, with no known systemic diseases, and she had no history of smoking. Her parents had experienced varying degrees of tooth loss.

**Figure 1.**
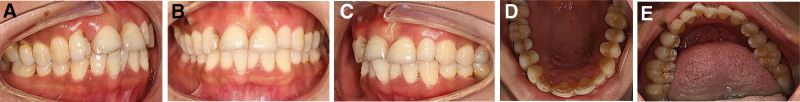
Pretreatment intraoral photographs. (A) Right side view. (B) Front view. (C) Left side view. (D) Upper occlusal view. (E) Lower occlusal view.

**Figure 2.**
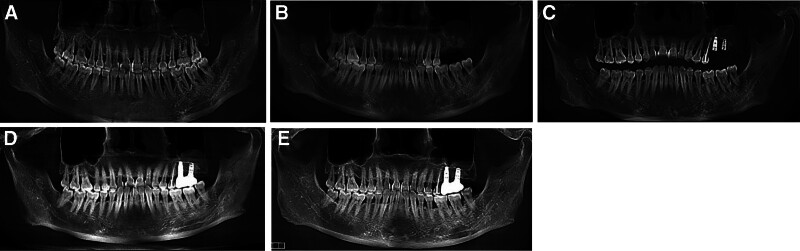
Panoramic radiograph. (A) Pretreatment. (B) Six months after periodontal surgery. (C) One year after periodontal surgery. (D) Three year after periodontal surgery. (E) Five year after periodontal surgery.

During the initial oral examination (Fig. 1), a significant amount of subgingival calculus was found in multiple sites. The dental plaque index was observed in 30.5% of the sites, with a periodontal probing depth (PPD) of ≥4 mm in 44.6% of sites and ≥6 mm in 15.5% of the sites. Additionally, bleeding on probing (BOP) was noted in 61.9% of the sites. Multiple teeth exhibited different degrees of mobility according to the Miller classification (class 0; 11 teeth, class 1; 7 teeth, class 2; 8 teeth, class 3; 2 teeth) (Table 1). The tooth no. 25 showing a wedge-shaped defect in the neck of the buccal surface of the crown, but the tooth did not respond to palpation or percussion, and did not respond to hypothermia testing. The surrounding soft tissues appeared normal with no signs of swelling or sinus tracts. Pretreatment panoramic radiographs (Fig. 2) revealed that the full mouth alveolar bone was absorbed to varying degrees into the middle 1/2 and apical 1/3 of the root, with teeth nos. 26, 27 absorbed into the apical region. Together with asymptomatic periapical radiolucent areas in tooth no. 25. The final diagnosis was generalized stage III grade C periodontitis, as per the classification of the American Academy of Periodontology.^[20]^ Tooth no. 25 was specifically diagnosed with asymptomatic apical periodontitis.After extensive communication and considering the patient’s strong refusal of orthodontic treatment and preference to preserve teeth, an interdisciplinary treatment plan was proposed and accepted.The Patient provided written informed consent for the publication of this case report and the accompanying images.

**Table 1:** Periodontal examinations at baseline.

Maxilla	17	16	15	14	13	12	11	21	22	23	24	25	26	27
PD buccal aspect 3 points (mm)	7 5 5	5 5 7	2 2 3	3 3 5	5 2 3	6 5 4	4 2 5	4 2 5	2 2 3	3 2 3	3 2 4	3 2 4	7 4 4	7 9 9
	+++	+++	+	++	+	++	+	++	+	+	+	++	+++	+++
GR buccal aspect 3 points (mm)	1 2 2	1 2 1	2 3 2	0 1 0	0 0 1	0 1 2	0 1 1	1 1 1	0 0 0	1 0 0	0 0 0	1 1 1	1 2 2	2 2 1
PD palatal aspect 3 points (mm)	6 3 5	7 2 7	3 3 2	4 3 3	4 4 6	4 4 4	5 2 3	3 2 4	2 3 2	3 3 3	3 3 3	3 3 5	9 4 6	7 3 3
	+++	+	+	+	++	++	++	++	+	++	+	+	+++	+++
GR palatal aspect 3 points (mm)	1 1 1	0 1 1	0 1 2	0 0 0	0 1 1	0 0 0	0 1 0	0 0 0	0 1 0	0 0 0	0 1 0	0 1 1	2 2 2	1 2 2
Miller grades of mobility	2	2	0	0	0	2	1	2	1	0	0	1	3	3
**Mandible**	**47**	**46**	**45**	**44**	**43**	**42**	**41**	**31**	**32**	**33**	**34**	**35**	**36**	**37**
PD lingual aspect 3 points (mm)	6 6 4	5 3 7	2 2 2	2 2 2	2 2 3	3 3 2	1 1 3	1 2 3	2 2 5	3 2 4	3 2 5	4 2 7	5 5 7	5 5 8
	++	+++	+	+	+	+		+	++	+	+++	+++	+++	+++
GR lingual aspect 3 points (mm)	1 1 0	0 1 1	0 0 0	0 0 0	1 1 1	1 0 0	0 2 1	0 0 0	1 0 0	0 0 0	1 2 1	0 1 1	1 2 1	1 1 1
PD buccal aspect 3 points (mm)	5 4 5	5 3 6	4 2 4	3 2 2	2 2 4	3 2 3	2 1 1	2 4 3	3 2 3	3 2 3	5 2 4	5 3 5	7 3 6	7 8 7
	++	+++	++	++	+	+		+	+	+	+++	+++	+++	+++
GR buccal aspect 3 points (mm)	0 1 1	1 2 1	1 1 0	1 1 0	1 2 1	0 0 0	1 2 1	0 0 0	0 1 0	1 2 1	0 1 0	0 1 0	1 1 1	1 2 2
Miller grades of mobility	1	1	0	1	0	0	0	0	1	0	2	2	2	2

Plaque index (30.5%).

+ = bleeding on probing, GR = gingival recession, PD = probing depth.

Bold values represent the serial numbers of different teeth.

The patient initially underwent nonsurgical periodontal treatment. After completion of the treatment, the patient showed poor compliance with the treatment plan. One year after frequent periodontal maintenance sessions, a reevaluation of clinical parameters showed significant improvement in periodontal inflammation compared to the first examination. However, there were still multiple teeth with vertical bone defects and residual periodontal pockets ≥ 5 mm (see Table S1, Supplemental Digital Content, https://links.lww.com/MD/P8, which revealed deep PPD in several areas with bleeding on probing). As a result, periodontal flap and tissue regeneration treatments were recommended for the 4 regions of the mouth (see Fig. S1, Supplemental Digital Content, https://links.lww.com/MD/P9, which demonstrates the need for periodontal flap and tissue regeneration treatments).

**Figure 3.**
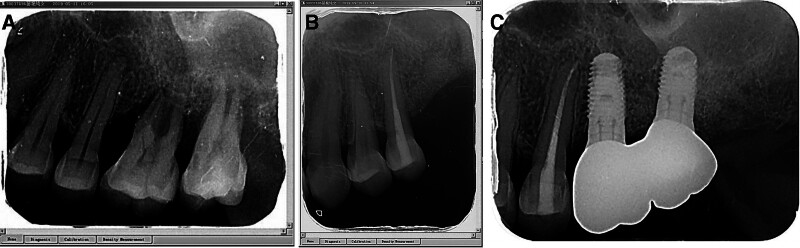
Dental radiograph of the left upper molar during root canal treatment and implant surgery. (A) Pretreatment. (B) Posttreatment of root canal treatment. (C) Five years after root canal treatment and implant surgery.

Due to the patient’s low medical compliance during the initial stages of treatment planning, the root canal treatment for tooth no. 25 was delayed and scheduled to take place 6 months after the periodontal surgical treatment. Following the root canal treatment, 2 implants (diameter * length, no. 26: 4.7 * 10 mm, no. 27: 4.7 * 8 mm, Zimmer Biomet, American, Warsaw, USA) were individually placed in the left upper molar position according to the implant surgical guide. The final zirconia crown restoration was completed 8 months after the implant placement (Fig. 3).

**Figure 4.**
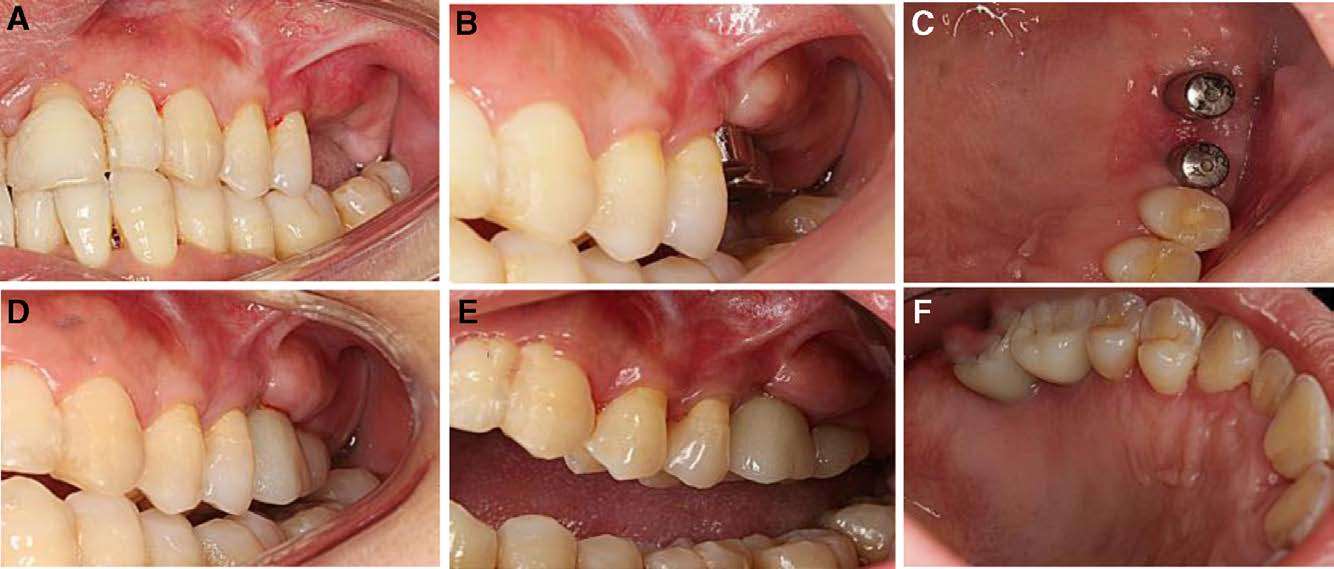
Intraoral photograph of the restoration loading and soft tissue augmentation procedure in the left upper molar position. (A) Suture removal 2 weeks after implant surgery. (B and C) Suture removal 2 weeks after restorative loading and a modified root-directional flap procedure (buccal and palatal). (D) Three years after final restoration. (E and F) Five years after final restoration (buccal and palatal).

After 4 months of implant placement, the width of the KM on the buccal side of the implant site was <2 mm. Restorative loading and a modified apically positioned flap procedure were performed at the implant site during the second stage of implant surgery. A curved incision was made approximately 2 to 3 mm lateral to the palate at the top of the alveolar ridge. The length of the incision was adjusted according to the diameter of the selected healing abutment. A buccal incision was made at the proximal and distal mesial ends of this incision and extended to about 2 mm above the root of the buccal gingival attachment, while preserving the gingival papillae of the adjacent teeth on both sides. The palatal incision was prepared with a partial-thickness flap up to the palatal end at the top of the alveolar ridge where the implant was positioned off-palatally, and then a full-thickness flap was prepared. After exposing the implant closure screw, the full-thickness flap was extended approximately 2 to 3 mm to the root of the buccal attachment gingiva to obtain a mixed tissue flap with a tip, which was then reset to the root direction. The cover screw was removed from the implant, a suitable healing abutment was selected and placed on the implant, followed by suturing and postoperative antimicrobial treatment (Fig. 4).

## 3. Results

**Figure 5.**
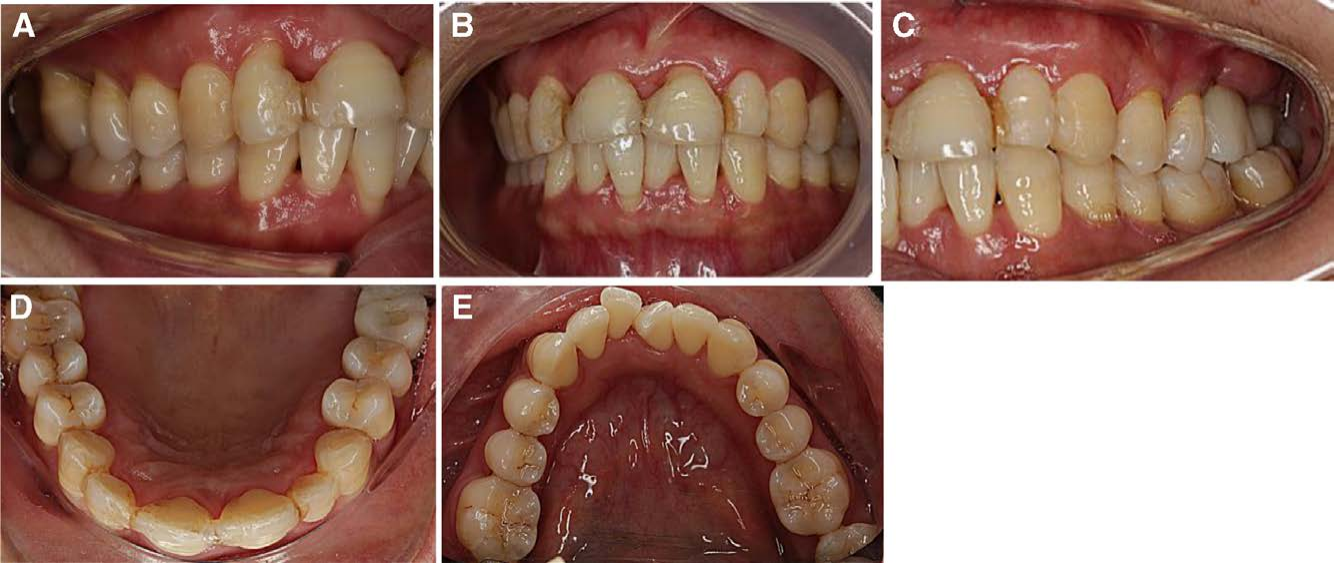
Intraoral photographs after 5 years of periodontal surgery. (A) Right side view. (B) Front view. (C) Left side view. (D) Upper occlusal view. (E) Lower occlusal view.

After consistent maintenance treatment, patients have successfully maintained a healthy and stable periodontal condition (Fig. 5).periodontal inflammation was effectively controlled, leading to a significant increase in bone density and alveolar bone regeneration in the anterior and posterior regions (Fig. 2). The dental plaque index was observed in 7.5% of the sites,and positive BOP sites and deep pockets ≥5 mm reduced notably (Table 2). The width of the buccal KM tissue at the implant site increased by 2 mm (Fig. 4).

**Table 2:** Periodontal examination 5 years after supportive periodontal therapy.

Maxilla	17	16	15	14	13	12	11	21	22	23	24	25	26	27
PD buccal aspect 3 points (mm)	3 1 2	3 3 2	1 3 3	2 3 2	2 1 2	2 2 2	3 3 3	3 3 3	1 2 1	3 2 3	2 3 2	3 1 2	–	–
GR buccal aspect 3 points (mm)	3 4 3	3 4 3	2 2 1	1 2 1	0 1 0	0 2 4	0 0 0	2 1 0	0 1 0	0 0 0	1 2 1	1 2 1		
PD palatal aspect 3 points (mm)	3 3 3	2 3 2	2 2 1	1 3 2	3 3 3	2 3 2	1 2 2	2 2 3	1 2 2	2 2 3	1 12	3 3 3	–	–
							+							
GR palatal aspect 3 points (mm)	2 2 2	1 2 2	1 2 1	1 1 0	0 0 0	1 1 1	0 0 1	1 0 0	1 1 1	0 0 0	0 0 1	0 1 0		
Miller grades of mobility	1	0	0	0	0	0	0	0	0	0	0	0	–	–
**Mandible**	**47**	**46**	**45**	**44**	**43**	**42**	**41**	**31**	**32**	**33**	**34**	**35**	**36**	**37**
PD lingual aspect 3 points (mm)	2 3 3	1 2 2	3 2 2	1 1 2	2 1 2	2 1 3	2 2 3	1 2 2	3 2 2	3 2 2	3 2 2	3 3 2	2 3 2	1 2 2
GR lingual aspect 3 points (mm)	0 1 1	0 1 0	1 1 1	0 0 0	0 0 1	0 1 0	1 1 0	0 0 0	0 0 1	0 0 0	0 1 1	1 1 1	0 1 1	2 1 0
PD buccal aspect 3 points (mm)	2 2 3	3 3 2	2 2 2	2 2 3	3 3 3	3 3 3	2 1 2	2 3 3	2 2 2	3 3 3	3 1 3	3 3 3	2 2 2	2 2 3
									+					
GR buccal aspect 3 points (mm)	1 2 1	2 2 2	2 3 2	0 1 0	1 2 1	0 0 0	3 4 3	0 1 0	1 2 2	0 1 0	1 2 1	1 2 2	1 2 2	2 2 2
Miller grades of mobility	0	0	0	0	0	0	0	0	0	0	0	0	0	0

Plaque index (7.5%).

+ = bleeding on probing, GR = gingival recession, PD = probing depth.

Bold values represent the serial numbers of different teeth.

## 4. Discussion

Patients with generalized stage III grade C periodontitis often present with dentition defects and malocclusion, necessitating a multidisciplinary approach to control inflammation and restore occlusal function. Effective plaque control not only resolves inflammation and promotes periodontal tissue health, but also reduces the risks associated with future implant and orthodontic procedures.^[21]^ In this case, a nonsurgical periodontal treatment plan was initiated, along with frequent periodontal maintenance sessions. Emphasis was placed on a home care program to effectively remove plaque microorganisms during initial and subsequent treatments.

After nonsurgical periodontal treatment, reevaluation revealed deep PPDs (>5 mm) in several areas with BOP, angular bone defects, and molars’ furcation involvement. Two consensus reports.^[22,23]^ From the AAP Regeneration Workshop have highlighted the effectiveness of periodontal regenerative therapies such as GTR, bone replacement grafting, and combination therapies for treating periodontal tissue defects. The initiation of surgical periodontal treatment offers benefits such as thorough debridement of inflammation from root surfaces and root bifurcations under visualization, restoration of periodontal hard and soft tissue morphology, and long-term stability through GTR combined with bone grafting.^[5]^ Teeth nos. 26 and 27 were minimally invasively extracted to prevent significant deficiencies in the alveolar ridges of the edentulous area. The bio-Oss bone powder was then implanted and covered with Bio-Gide membranes to restore alveolar ridge dimensions, maintain implant stability, and reconstruct normal occlusal function.^[6]^ Following significant improvement in periodontal inflammation and tooth loosening, the patient’s compliance increased, leading to smoother doctor–patient communication and treatment.

The thickness and width of peri-implant KM are closely related to long-term implant stability. Studies by Gharpure et al^[24]^ and Thoma et al^[25]^ have shown that peri-implant sites with KM thickness and width ≤2 mm are more likely to lead to brushing discomfort and plaque accumulation, which can contribute to the development of peri-implantitis. Therefore, proper management of peri-implant soft tissues is essential for preventing peri-implantitis. Numerous studies have shown that the width of peri-implant KM can be increased through techniques such as apically positioned flaps or vestibuloplasty,often in combination with free gingival grafts or allograft materials.^[26]^ Additionally, increasing the thickness of peri-implant KM with subepithelial connective tissue grafts or soft tissue replacement grafts.^[27]^ A systematic review has shown that apically positioned flap significantly reduced probing depth and plaque index, regardless of the type of soft tissue graft material used, making it one of the most effective methods for increasing the width of KM.^[14]^

Soft tissue augmentation procedures at the dental implant site can be performed at various time points, including pre-implant surgery, concurrent with implant surgery, during the second stage of implant surgery, and post-implant loading.^[28]^ Bassetti et al^[15]^ highlighted the significance of soft tissue remodeling during the second stage of implant surgery. In this case, a modified apically positioned flap procedure was carried out concurrently with the second stage of implant surgery. This displacement resulted in an expansion of the width of the buccal KM around the implant. The technique offers several advantages, including simplification of the procedure, elimination of the need for a second surgical site, and reduced trauma and discomfort for the patient. Partial-full thickness flaps exhibit favorable hematological properties, high survival rates, resistance to infection, a color and appearance similar to adjacent gingiva, minimal changes in anatomical morphology on the labial and buccal sides, and significantly reduced shrinkage rates.The 5-year follow-up after implant loading demonstrated a stable position of the implant gingival margin and healthy gingival tissue. This technique has shown positive results in KM reconstruction and offers a valuable technical approach for patients with inadequate width of peri-implant KM. Nevertheless, additional analysis is required regarding specific case characteristics and surgical procedures.

The combined multidisciplinary treatment model is gaining increasing attention in the management of periodontitis, bringing together expertise from different disciplines such as periodontics, implantology, orthodontics, and prosthodontics. Nonsurgical periodontal treatment focuses on reducing inflammation and restoring health by creating a compatible root surface.^[29]^ While mechanical debridement has limitations, its combination with newer biologics is improving outcomes. Azithromycin microspheres help with inflammation and tissue repair, and melatonin improves periodontal health.^[30,31]^ Natural ingredients such as polyphenols and alkaloids provide anti-inflammatory benefits and combat drug-resistant bacteria.^[32]^ Studies have confirmed that innovative materials such as nanomaterials, bioceramics, and functional hydrogels are effective in treating periodontitis.^[33–35]^ Future research should focus on overcoming the challenges of clinical application, particularly in developing new materials that ensure biosafety, controlled drug release, and inflammation modulation.

Integrating periodontal and orthodontic therapies in the management of periodontitis effectively improves anterior tooth displacement by reducing inflammatory markers such as NOD-like receptor protein 3 inflammasomes and high mobility group protein B1, facilitating bone repair, and ensuring long-term periodontal stability.^[36,37]^ Controlling inflammation prior to orthodontic treatment is critical because tooth movement is safe during periods of stability but can exacerbate tissue damage during inflammation. Importantly, tooth movement helps reposition plaque, promotes tissue regeneration, and optimizes implant space.^[38]^ The success of implant restorations is closely linked to a patient’s periodontal health. Research shows that in patients with severe periodontitis, systemic periodontal treatment can result in a 23.2% increase in implant bone volume.^[39]^ However, the high rates of peri-implantitis (9.1%) and peri-implant mucositis (76.2%) highlight the importance of timing implant placement and managing risk factors such as plaque control and keratinized tissue width.^[40]^ Multidisciplinary combination therapy is essential to manage complex dental cases.^[41]^ Importantly, periodontitis has a bidirectional relationship with systemic diseases such as diabetes and cardiovascular problems, involving various inflammatory and immune pathways.^[42,43]^ The effective management of periodontitis requires a holistic approach that considers both systemic and oral health. This includes creating a personalized treatment plan to improve outcomes and overall well-being. A multidisciplinary strategy addresses complex oral issues while ensuring long-term maintenance of oral function and aesthetics.

## 5. Conclusion

The multidisciplinary surgical approach demonstrated efficacy in restoring periodontal health, reconstructing occlusal function, enhancing psychological well-being, and offering valuable insights into the management of severe periodontitis. In the second stage of implant surgery, a modified apically positioned flap procedure may serve as an option for soft tissue augmentation at the dental implant site.

## Author contributions

**Data curation:** Shun Mao, Fei Ma, Chang Zeng, Yan Xie.

**Funding acquisition:** Hui Xie.

**Methodology:** Yan Xie.

**Supervision:** Hui Xie, Jincai Guo.

**Writing – original draft:** Shun Mao, Jian Liu.

**Writing – review & editing:** Shun Mao.

## Supplementary Material



## References

[1] Agudio G Buti J Bonaccini D Pini Prato G Cortellini P. Longevity of teeth in patients susceptible to periodontitis: clinical outcomes and risk factors associated with tooth loss after active therapy and 30 years of supportive periodontal care. J Clin Periodontol. 2023;50:520–32. 36631984 10.1111/jcpe.13770

[2] Needleman I Almond N Leow N Phillips J. Outcomes of periodontal therapy: Strengthening the relevance of research to patients. A co-created review. Periodontol 2000. 2023;14:1–15. 10.1111/prd.12483 36786482

[3] Kwon T Lamster IB Levin L. Current concepts in the management of periodontitis. Int Dent J. 2021;71:462–76. 34839889 10.1111/idj.12630 PMC9275292

[4] Sanz M Herrera D Kebschull M. EFP Workshop participants and methodological consultants. Treatment of stage I-III periodontitis-The EFP S3 level clinical practice guideline. J Clin Periodontol. 2020;47:4–60. 32383274 10.1111/jcpe.13290 PMC7891343

[5] Majzoub J Barootchi S Tavelli L Wang CW Chan HL Wang HL. Guided tissue regeneration combined with bone allograft in infrabony defects: clinical outcomes and assessment of prognostic factors. J Periodontol. 2020;91:746–55. 31680235 10.1002/JPER.19-0336

[6] Amaral Valladão CA Jr Freitas Monteiro M Joly JC. Guided bone regeneration in staged vertical and horizontal bone augmentation using platelet-rich fibrin associated with bone grafts: a retrospective clinical study. Int J Implant Dent. 2020;6:72. 33067730 10.1186/s40729-020-00266-y PMC7567776

[7] Dias AT de Menezes CC Kahn S Fischer RG da Silva Figueredo CM Fernandes GVO. Gingival recession treatment with enamel matrix derivative associated with coronally advanced flap and subepithelial connective tissue graft: a split-mouth randomized controlled clinical trial with molecular evaluation. Clin Oral Investig. 2022;26:1453–63. 10.1007/s00784-021-04119-9 34536136

[8] Polymeri A Loos BG Aronovich S Steigmann L Inglehart MR. Risk factors, diagnosis, and treatment of peri-implantitis: a cross-cultural comparison of U.S. and European periodontists’ considerations. J Periodontol. 2022;93:481–92. 34390497 10.1002/JPER.21-0010 PMC10138758

[9] Herrera D Berglundh T Schwarz F. Prevention and treatment of peri-implant diseases-The EFP S3 level clinical practice guideline. J Clin Periodontol. 2023;50:4–76. 10.1111/jcpe.13823 37271498

[10] Dos Santos Martins BG Fernandes JCH Martins AG de Moraes Castilho R de Oliveira Fernandes GV. Surgical and nonsurgical treatment protocols for peri-implantitis: an overview of systematic reviews. Int J Oral Maxillofac Implants. 2022;37:660–76. 35904822 10.11607/jomi.9659

[11] Oliveira CAB Pereira VL Dos Santos JN Araujo NS Cury PR. Influence of keratinized mucosa on peri-implant diseases: a retrospective cohort study in humans. Oral Maxillofac Surg. 2024;28:331–6. 36847879 10.1007/s10006-023-01144-8

[12] Mahardawi B Jiaranuchart S Damrongsirirat N. The lack of keratinized mucosa as a risk factor for peri-implantitis: a systematic review and meta-analysis. Sci Rep. 2023;13:3778. 36882495 10.1038/s41598-023-30890-8 PMC9992510

[13] Ramanauskaite A Schwarz F Sader R. Influence of width of keratinized tissue on the prevalence of peri-implant diseases: a systematic review and meta-analysis. Clin Oral Implants Res. 2022;33:8–31. 35763022 10.1111/clr.13766

[14] Tavelli L Barootchi S Avila-Ortiz G Urban IA Giannobile WV Wang HL. Peri-implant soft tissue phenotype modification and its impact on peri-implant health: a systematic review and network meta-analysis. J Periodontol. 2021;92:21–44. 32710810 10.1002/JPER.19-0716

[15] Bassetti RG Stähli A Bassetti MA Sculean A. Soft tissue augmentation procedures at second-stage surgery: a systematic review. Clin Oral Investig. 2016;20:1369–87. 10.1007/s00784-016-1815-2 27041111

[16] de Siqueira GRC Tavares JR Pedrosa RF de Siqueira RAC Fernandes GVO. Immediate implant with provisionalization and soft tissue grafting after 4-year follow-up. Clin Adv Periodontics. 2022;12:32–8. 33914411 10.1002/cap.10162

[17] Rondone EM Leitão-Almeida B Pereira MS Fernandes GVO Borges T. The use of tissue grafts associated with immediate implant placement to achieve better peri-implant stability and efficacy: a systematic review and meta-analysis. J Clin Med. 2024;13:821. 38337515 10.3390/jcm13030821 PMC10856075

[18] Martins SCR Marques MDC Vidal MG. Is the facial bone wall critical to achieving esthetic outcomes in immediate implant placement with immediate restoration? A systematic review. Adv Clin Exp Med. 2024;33:979–97. 38180330 10.17219/acem/173573

[19] Kuebler A Noelken R. The influence of connective tissue grafting on the reconstruction of a missing facial bone wall using immediate implant placement and simultaneous bone reconstruction: a retrospective long-term cohort study. Int J Implant Dent. 2024;10:25. 38760582 10.1186/s40729-024-00533-2 PMC11101404

[20] Caton JG Armitage G Berglundh T. A new classification scheme for periodontal and peri-implant diseases and conditions – introduction and key changes from the 1999 classification. J Periodontol. 2018;89:S1–8. 29926946 10.1002/JPER.18-0157

[21] Erbe C Heger S Kasaj A Berres M Wehrbein H. Orthodontic treatment in periodontally compromised patients: a systematic review. Clin Oral Investig. 2023;27:79–89. 10.1007/s00784-022-04822-1 PMC9877066 36502508

[22] Reynolds MA Kao RT Camargo PM. Periodontal regeneration – intrabony defects: a consensus report from the AAP Regeneration Workshop. J Periodontol. 2015;86:S105–7. 25315019 10.1902/jop.2015.140378

[23] Reddy MS Aichelmann-Reidy ME Avila-Ortiz G. Periodontal regeneration – furcation defects: a consensus report from the AAP Regeneration Workshop. J Periodontol. 2015;86:S131–3. 25644296 10.1902/jop.2015.140379

[24] Gharpure AS Latimer JM Aljofi FE Kahng JH Daubert DM. Role of thin gingival phenotype and inadequate keratinized mucosa width (<2 mm) as risk indicators for peri-implantitis and peri-implant mucositis. J Periodontol. 2021;92:1687–96. 33856690 10.1002/JPER.20-0792

[25] Thoma DS Benić GI Zwahlen M Hämmerle CH Jung RE. A systematic review assessing soft tissue augmentation techniques. Clin Oral Implants Res. 2009;20:146–65. 19663961 10.1111/j.1600-0501.2009.01784.x

[26] Sculean A Gruber R Bosshardt DD. Soft tissue wound healing around teeth and dental implants. J Clin Periodontol. 2014;41:S6–22. 24641001 10.1111/jcpe.12206

[27] Thoma DS Naenni N Figuero E. Effects of soft tissue augmentation procedures on peri-implant health or disease: a systematic review and meta-analysis. Clin Oral Implants Res. 2018;29:32–49. 29498129 10.1111/clr.13114

[28] Thoma DS Cosyn J Fickl S. working group 2 of the 6th EAO Consensus Conference 2021. Soft tissue management at implants: summary and consensus statements of group 2. The 6th EAO Consensus Conference 2021. Clin Oral Implants Res. 2021;32:174–80. 34145925 10.1111/clr.13798 PMC8596754

[29] Cobb CM Sottosanti JS. A re-evaluation of scaling and root planing. J Periodontol. 20211378;92:1370. 33660307 10.1002/JPER.20-0839

[30] Ouyang Z Chen X Wang Z. Azithromycin-loaded PLGA microspheres coated with silk fibroin ameliorate inflammation and promote periodontal tissue regeneration. Regen Biomater. 2024;12:rbae146. 39791015 10.1093/rb/rbae146 PMC11717352

[31] Purpura S Fernandes GVO Oliveira FP de Castro FC. Effects of melatonin in the non-surgical treatment of periodontitis: a systematic review. Appl Sci. 2022;12:11698.

[32] Hashim NT Babiker R Rahman MM. Natural bioactive compounds in the management of periodontal diseases: a comprehensive review. Molecules. 2024;29:3044. 38998994 10.3390/molecules29133044 PMC11242977

[33] Li H Zhang D Bao P. Recent advances in functional hydrogels for treating dental hard tissue and endodontic diseases. ACS Nano. 2024;18:16395–412. 38874120 10.1021/acsnano.4c02754

[34] Wang D Li Q Xiao C Wang H Dong S. Nanoparticles in periodontitis therapy: a review of the current situation. Int J Nanomedicine. 2024;19:6857–93. 39005956 10.2147/IJN.S465089 PMC11246087

[35] Sharma P Saurav S Tabassum Z. Applications and interventions of polymers and nanomaterials in alveolar bone regeneration and tooth dentistry. RSC Adv. 2024;14:36226–45. 39534053 10.1039/d4ra06092j PMC11555558

[36] Liu Y Shi X Lin G Guo N. Effects of periodontal initial therapy combined with orthodontic treatment on anterior tooth function and inflammatory factors in gingival crevicular fluid in patients with periodontal disease induced anterior tooth displacement. Pak J Med Sci. 2023;39:1620–5. 37936736 10.12669/pjms.39.6.7135 PMC10626121

[37] Yu L Zhou C Wei Z Shi Z. Effect of combined periodontal-orthodontic treatment on NOD-like receptor protein 3 and high mobility group box-1 expressions in patients with periodontitis and its clinical significance. Medicine (Baltim). 2019;98:e17724. 10.1097/MD.0000000000017724 PMC6946199 31689812

[38] Antonarakis GS Zekeridou A Kiliaridis S. Giannopoulou C. Periodontal considerations during orthodontic intrusion and extrusion in healthy and reduced periodontium. Periodontol 2000. 2024;3:1–25. 10.1111/prd.12578 38831560

[39] Kubica H Smektała T Owczarek AJ Sporniak-Tutak K Bednarz W. Computer-guided immediate full-arch implant reconstruction of the mandible in severe periodontally compromised patients: three-dimensional analysis of bone volume changes. Int J Oral Maxillofac Implants. 2022;37:346–55. 35476864 10.11607/jomi.9072

[40] Lv PX Zhong JS Ouyang XY Iao S Liu J Xie Y. Investigation of peri-implant diseases prevalence and related risk indicators in patients with treated severe periodontitis over 4 years after restoration. J Dent Sci. 2024;19:894–9. 38618128 10.1016/j.jds.2023.08.010 PMC11010623

[41] Kahn S Dias AT Nobre V de Oliveira LZ Fernandes GVO. Endodontic and periodontal treatment of complete buccal root and apex exposition: a challenging case report with 17 months follow-up. Clin Adv Periodontics. 2022;12:152–8. 34162015 10.1002/cap.10173

[42] Tonetti MS Van Dyke TE; Working group 1 of the joint EFP/AAP workshop. Periodontitis and atherosclerotic cardiovascular disease: consensus report of the Joint EFP/AAP workshop on periodontitis and systemic diseases. J Clin Periodontol. 2013;40:S24–9. 23627332 10.1111/jcpe.12089

[43] Mata-Monterde M Serrano-Valcarce A Almiñana-Pastor PJ Micó-Martínez P López-Roldán A. miRNAs as epigenetic biomarkers in the study of the bidirectional relationship between Type 2 diabetes mellitus and periodontitis: a systematic review. Int J Mol Sci . 2024;25:10723. 39409052 10.3390/ijms251910723 PMC11477124

