# Supplementary material for: Multidisciplinary treatment combined with a modified apically positioned flap technique for generalized stage III grade C periodontitis: A five-year follow-up case report
Source: Medicine (Baltimore). 2025 May 23;104(21):e42037. doi: 10.1097/MD.0000000000042037 (PMC12113923; doi:10.1097/MD.0000000000042037)
Supplement: Supplementary file 1 [file medi-104-e42037-s001.pdf]

Supplemental Table 1. Periodontal examination 1 yr after initial non-surgical periodontal treatment

**Maxilla**	**17**	**16**	**15**	**14**	**13**	**12**	**11**	**21**	**22**	**23**	**24**	**25**	**26**	**27**
PD buccal aspect 3 points (mm)	6 5 6<br>+ +	5 4 5<br>+ ++	4 2 2	3 2 3<br>+	4 3 3	5 5 5<br>+ +	3 3 3	4 3 4	2 2 2	3 2 2	3 2 3	3 3 3<br>+	6 5 4<br>+ +	5 7 8<br>+ ++
GR buccal aspect 3 points (mm)	2 3 2	2 3 2	1 3 1	1 2 0	0 0 0	0 1 3	0 0 0	2 3 2	1 2 1	0 0 0	1 1 1	1 1 0	3 3 4	4 4 3
PD palatal aspect 3 points (mm)	5 5 3<br>+ ++	5 3 7<br>+ +	3 3 2	3 3 3	3 4 3	4 3 4	4 3 3	3 4 4	2 3 1	3 3 3	2 3 3	3 4 5<br>+	7 5 6<br>+ +	6 4 3<br>+
GR palatal aspect 3 points (mm)	1 2 1	1 1 1	0 1 1	1 1 0	0 0 0	0 0 1	0 1 0	0 0 0	0 0 0	0 1 0	0 0 1	1 1 1	1 2 2	3 3 2
Miller grades of mobility	2	1	0	0	0	2	0	1	0	0	0	1	3	3
**Mandible**	**47**	**46**	**45**	**44**	**43**	**42**	**41**	**31**	**32**	**33**	**34**	**35**	**36**	**37**
PD lingual aspect 3 points (mm)	3 4 3	4 3 6<br>+ +	2 3 3	3 2 2	3 1 3	3 3 3	2 3 3	2 2 2<br>+ +	3 2 4	4 2 4	2 2 5<br>+ +	3 3 7<br>+	4 5 5<br>++	4 5 6<br>+
GR lingual aspect 3 points (mm)	0 2 1	0 0 1	0 0 0	0 1 1	1 1 0	0 0 0	0 2 1	0 0 0	0 1 0	0 0 0	1 0 0	0 0 0	1 1 0	1 2 1
PD buccal aspect 3 points (mm)	4 3 4	3 3 6<br>+ +	3 3 1	3 3 3	3 2 2	1 2 3	2 2 2	1 2 2	2 3 4	3 3 2	3 3 4<br>+	4 4 4<br>++	4 3 5<br>+ +	6 4 6<br>+ +
GR buccal aspect 3 points (mm)	0 1 1	1 1 1	1 2 1	1 2 0	0 1 0	0 0 0	2 3 2	0 0 0	1 1 1	0 1 0	0 0 0	0 1 1	2 2 1	1 1 1
Miller grades of mobility	0	0	0	0	0	0	0	0	0	0	1	2	1	2

Plaque index(12.5%), PD= Probing depth, + = Indicates bleeding on probing, GR= gingival recession.
