# Supplementary material for: Multidisciplinary treatment combined with a modified apically positioned flap technique for generalized stage III grade C periodontitis: A five-year follow-up case report
Source: Medicine (Baltimore). 2025 May 23;104(21):e42037. doi: 10.1097/MD.0000000000042037 (PMC12113923; doi:10.1097/MD.0000000000042037)
Supplement: Supplementary file 2 [file medi-104-e42037-s002.pdf]

Supplementary Figure 1A to 1Q. Oral photographs of the periodontal surgery. 1A: Teeth nos. 11, 12, 13, 21, 22, and 23 were temporarily splinted using bonding resin, and appropriate occlusal adjustments were performed; 1B-1E: Teeth nos. 26 and 27 were extracted, and the alveolar socket underwent debridement , guided bone regeneration was performed simultaneously; 1F-1Q: Guided tissue regeneration combined with bone allograft was carried out on the bone defect areas of teeth nos. 12, 14, 15, 16, 17, 21, 34, 35, 36, 37, and 46.

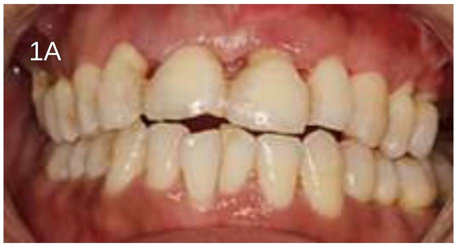

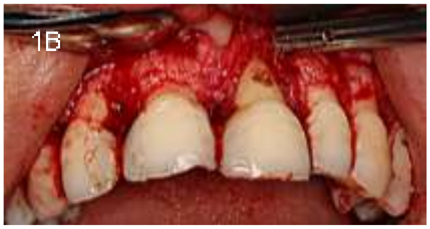

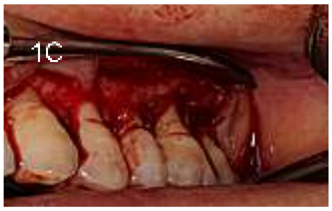

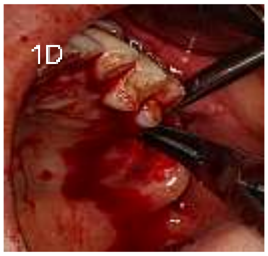

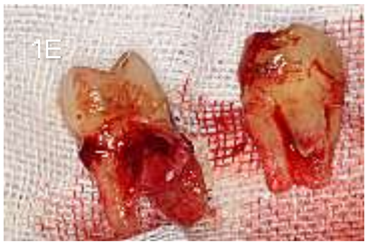
1E

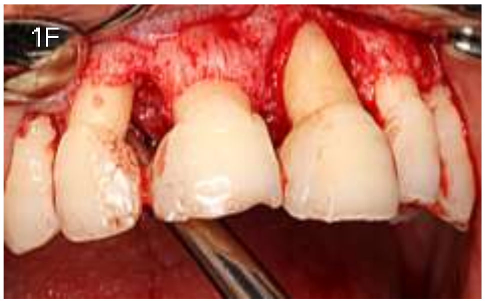
1F

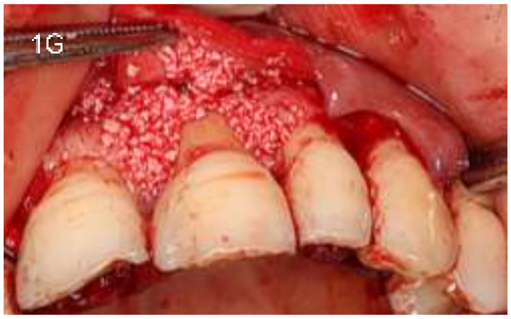
1G

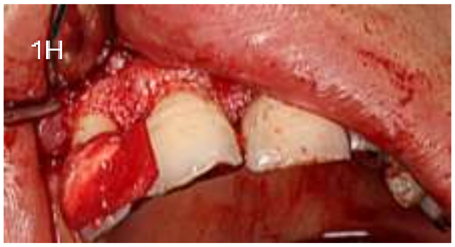
1H

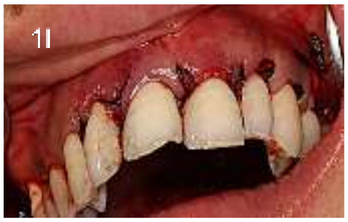
1I

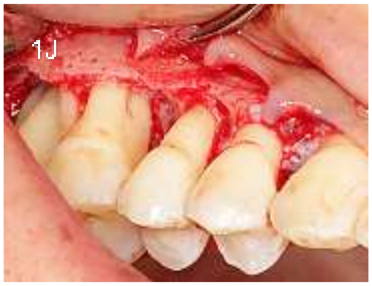
1J

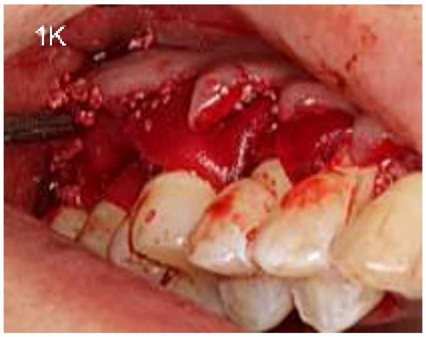
1K

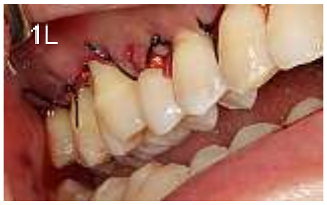
1L

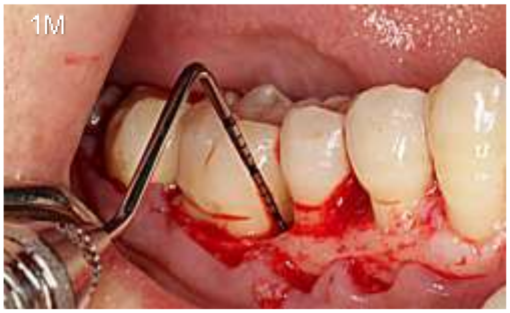
1M

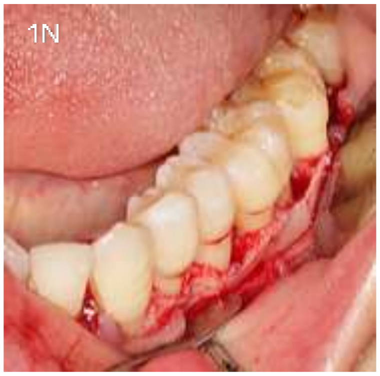
1N

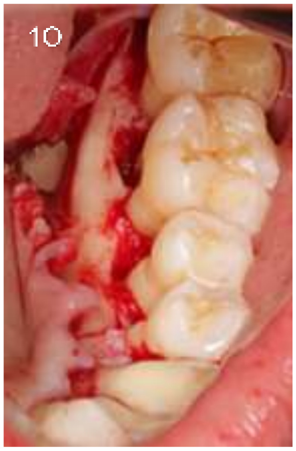
1O

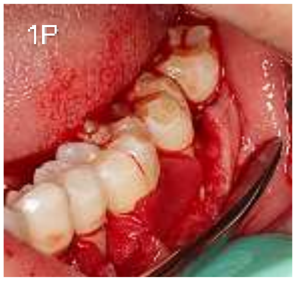
1P

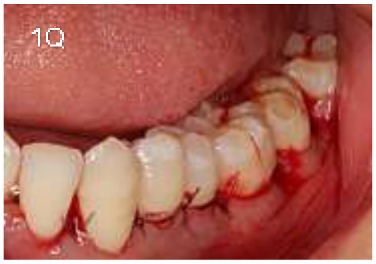
1Q
